# Additions and corrections to the check list of the Noctuoidea (Insecta, Lepidoptera) of North America north of Mexico III

**DOI:** 10.3897/zookeys.527.6151

**Published:** 2015-10-15

**Authors:** J. Donald Lafontaine, B. Christian Schmidt

**Affiliations:** 1 Canadian National Collection of Insects, Arachnids, and Nematodes, Biodiversity Program, Agriculture and Agri-Food Canada, K.W. Neatby Bldg., 960 Carling Ave., Ottawa, Ontario, Canada K1A 0C6

**Keywords:** Canada, United States, Noctuoidea, Erebidae, Eulepidotinae, Noctuidae, Dyopsinae, *Litoprosopus*

## Abstract

A total of 124 additions and corrections are listed and discussed for the check list of the Noctuoidea of North America north of Mexico published in 2010. Twenty-eight species are added to the list, 16 through new species descriptions, eight as a result of taxonomic splits, and four based on newly recorded species. Forty-eight species are deleted from the list, 41 through synonymy, and seven that were based on misidentifications. Twelve changes are corrections in the spelling of names, or changes in parentheses on dates of publication. Twenty-seven are changes in taxonomy of names where no species are added or deleted; eight changes involve the renumbering of existing species for better taxonomic arrangement. Within the text 2 **stat. n.**, 10 **stat. rev.**, 27 **syn. n.**, 5 **syn. rev.**, and 1 **comb. n.** are proposed for the first time.

## Introduction

Continuing work on the taxonomy and systematics of New World Noctuoidea has resulted in 124 additional changes to the check list of North American Noctuoidea (Lafontaine and Schmidt 2010). These are in addition to the 115 changes made to the list in in 2011 (Lafontaine and Schmidt 2011) and 64 changes made in 2013 (Lafontaine and Schmidt 2013). The new total of Noctuoidea species in North America north of Mexico is 3672 species.

## Materials and methods

### Repository abbreviations

Taxonomic changes are based on examination of material, especially type specimens, in the following collections:

AMNH The American Museum of Natural History, New York, NY, USA

ANSP The Academy of Natural Sciences, Philadelphia, Pennsylvania, USA

BMNH The Natural History Museum [statutorily: British Museum (Natural History)], London, UK

CNC Canadian National Collection of Insects, Arachnids, and Nematodes, Ottawa, Ontario, Canada

CUIC Cornell University Insect Collection, Ithaca, New York, USA

MNHN Muséum National d’ Histoire Naturelle, Paris, France

TLSC Texas Lepidoptera Survey Collection, Houston, Texas, USA

JTTC James T. Troubridge Collection, Hagarsville, Ontario, Canada

USNM National Museum of Natural History [formerly, United States National Museum], Washington, District of Columbia, USA

## Results

**Corrections, additions, and changes** (highlighted in **bold**)

p. 3 & p. 42 **Subfamily Dyopsinae Guenée, 1852** [insert after Family Noctuidae before Subfamily Plusiinae]

930098 *Schizura****ipomaeae*** Doubleday, 1841

930139 **Delete***Scotura annulata* (Guérin-Méneville, 1844)

930142 *Gynaephora rossii* (Curtis, 1835)

930196 **Delete***Propyria schausi* (Dyar, 1898)

930217 ***Manulea****bicolor* (Grote, 1864)

930227.1 ***Crambidia xanthocorpa* Lewis, 2014**

930314 *Spilosoma pteridis* H. Edwards, 1875

**syn. *Spilosoma danbyi* (Neumögen & Dyar, 1893)**

930350 ***Hypocrita celina* (Boisduval, 1870)**

**syn.***Hypocrita escuintla* (Schaus, 1920)

930358 *Dysschema howardi* (H. Edwards, 1887)

930392 **Delete***Neritos prophaea* (Schaus, 1905)

930402 *Cycnia collaris* (Fitch, 1856)

**syn. *Cycnia inopinatus* (H. Edwards, 1882)**

**syn. *Cycnia tenerosa* (Dyar, 1913)**

930440 *Cisseps fulvicollis* (Hübner, [1818])

**syn. *Cisseps packardii* (Grote, 1865)**

**syn. *Cisseps wrightii* (Stretch, 1885)**

930445 *Eucereon****erythrolepsis*** Dyar, 1910

930457 **Delete***Cosmosoma teuthras* (Walker, 1854)

**930462.1 *Syntomeida syntomoides* (Boisduval, 1836)**

930581 ***Hypena tenebrosa* (Walker, 1865)**

**syn. *Hypena annulalis* (Grote, 1876)**

930604 ***Rusicada****privata* (Walker, 1865)

930605 *Anomis impasta***of authors, not Guenée, 1852**

930616 *Gonodonta***fulvangula** Geyer, 1832

930631 *Psammathodoxa cochlidioides* Dyar, 1921

**syn. *Psammathodoxa calligrapha* (Hampson, 1926)**

930703 *Hemeroplanis reversalis* (Smith, 1907)

**syn. *Hemeroplanis cuernavacalis* (Dyar, 1912)**

930710 *Hemeroplanis immaculalis* (Harvey, 1875)

**syn. *Hemeroplanis obliqualis* (H. Edwards, 1886)**

930727 *Hyperstrotia nana* (Hübner, 1818)

**syn. *Hyperstrotia villificans* (Barnes & McDunnough, 1918)**

**930769.1 *Catocala myristica* Kons & Borth, 2015**

**930846.1 *Catocala aestivalia* Kons & Borth, 2015**

930907 *Drasteria maculosa* (Behr, 1870)

**syn. *Drasteria nubicola* (Behr, 1870)**

**930925.1 *Doryodes desoto* Lafontaine & Sullivan, 2015**

**930925.2 *Doryodes okaloosa* Sullivan & Lafontaine, 2015**

930927 *Doryodes spadaria* Guenée, [1858]

**syn. Doryodes spadaria race grandipennis Barnes & McDunnough, 1918**

**930927.1 *Doryodes fusselli* Sullivan & Lafontaine, 2015**

**930927.2 *Doryodes latistriga* Sullivan & Lafontaine, 2015**

**930927.3 *Doryodes broui* Lafontaine & Sullivan, 2015**

**930927.4 *Doryodes reineckei* Sullivan & Lafontaine, 2015**

**930970.1 *Lesmone aenaria* (Druce, 1890)**

**syn. *Lesmone lampea* (Druce, 1890)**

**syn. *Lesmone detrahens* of authors, not (Walker, 1858)**

930982 *Heteranassa mima* (Harvey, 1876)

**syn. *Heteranassa fraterna* (Smith, 1899)**

**syn. *Heteranassa minor* (Smith, 1899)**

**931000 *Toxonprucha excavata* (Walker, 1865)**

**syn. *Toxonprucha minuscula* (Walker, 1865)**

**syn. *Toxonprucha amoena* Möschler, 1890**

**syn. *Toxonprucha diffundens* of authors, not (Walker, 1858)**

**931018 *Zale viridans* of authors, not (Guenée, 1852)**

**931019 Delete***Zale strigimacula* (Guenée, 1852)

931062 **Delete***Amolita sentalis* (Kaye, 1901)

**931096 –931100 Move***Litoprosopus* spp. **to Subfamily Dyopsinae as 931160.1 –931160.5**

931111 *Paectes abrostoloides* (Guenée, 1852)

**syn. *Paectes delineata* (Guenée, 1852)**

931156 *Collomena****inflexa* (Morrison, 1875)**

**syn. *Collomena filifera* of authors, not (Walker, 1857)**

**931160.1***Litoprosopus futilis* (Grote & Robinson, 1868)

**931160.2***Litoprosopus hatuey* (Poey, 1832)

syn. *Litoprosopus haitiensis* of authors, not Hampson, 1926

**931160.3***Litoprosopus bahamensis* Hampson, 1926

**931160.4***Litoprosopus coachella* Hill, 1921

**931160.5***Litoprosopus confligens* (Walker, [1858])

931253 ***Amyna stricta* (Walker, 1858)**

**syn. *Amyna axis* of authors, not Guenée, 1852**

**syn. *Amyna octo* of authors, not Guenée, 1852**

**syn. *Amyna flaviguttata* (Walker, 1858)**

**syn. *Amyna orbica* (Morrison, 1874)**

**syn. *Amyna tecta* (Grote, 1876)**

**931253.2 *Concana mundissima* Walker, [1858**]

**931254.1 *Cydosia majuscula* (H. Edwards, 1881)**

**931257.1 *Tripudia inquaesita* (Barnes & Benjamin, 1924)**

931412 *Raphia frater* Grote, 1864

ssp. frater Grote, 1864

syn. *Raphia personata* (Walker, 1865)

**ssp. abrupta Grote, 1864**

syn. *Raphia flexuosa* (Walker, 1865)

**ssp. coloradensis Putnam-Cramer, 1886**

**syn. *Raphia pallula* H. Edwards, 1886**

**ssp. piazzi Hill, 1927**

**ssp. cinderella Smith, 1903**

**ssp. elbea Smith, 1908**

**931442.1 *Acronicta fallax* (Herrich-Schäffer, 1854)**

**syn. *Acronicta geminata* (Smith, 1903)**

**931477.1 *Acronicta menyanthidis* (Vieweg, 1790)**

931496 ***Chloronycta****tybo* (Barnes, 1904)

931550 *Psaphida rolandi* (Grote, 1874)

931564 *Feralia comstocki* Grote, 1874

931606 *Emarginea percara* (Morrison, 1875)

**syn. *Emarginea pallida* (Smith, 1902)**

931642 *Cropia connecta* (Smith, 1894)

**syn. *Cropia templada* (Schaus, 1906)**

**931643.1 *Cropia indica* (Walker, [1858])**

931767 *Catabenoides vitrina* (Walker, 1857)

**syn. *Catabenoides divisa* (Herrich-Schäffer, 1868)**

931773 *Pseudacontia crustaria* (Morrison, 1875)

**syn. *Pseudacontia cansa* Smith, 1908**

**syn. *Pseudacontia louisa* Smith, 1908**

931784 *Stylopoda groteana* (Dyar, 1903)

**syn. *Stylopoda anxia* Smith, 1908**

**931906.1 *Sympistis forbesi* Zacharczenko & Wagner, 2014**

**932022.1 *Ogdoconta margareta* Crabo, 2015**

932036 *Heliothodes****diminutivus*** (Grote, 1873)

syn. ***Heliothodes fasciata*** (H. Edwards, 1875)

932044 *Psectrotarsia hebardi* (Skinner, 1917)

932054 ***Chloridea****virescens* (Fabricius, 1777)

932055 ***Chloridea****subflexa* (Guenée, 1852)

932223 *Spodoptera eridania* (**Stoll, 1782**)

**932236.1 *Elaphria trolia* (Dyar, 1914)**

932278 ***Acroria pulchra* (Möschler, 1886)**

**syn. *Acroria terrens* of authors, not Walker, 1857**

932284 *Nedra stewarti* (Grote, 1875)

**syn. *Nedra dora* Clarke, 1940**

**932361.1 *Cherokeea attakullakulla* Sullivan & Quinter, 2014**

932480 *Papaipema insulidens* (Bird, 1902)

**syn. *Papaipema birdi* (Dyar, 1908)**

**syn. *Papaipema pertincta* Dyar, 1920**

932483 *Papaipema harrisii* (Grote, 1881)

**syn. *Papaipema verona* (Smith, 1899)**

932552 *Lithophane viridipallens* Grote, 1877

**syn. *Lithophane pruena* (Dyar, 1910)**

932634 *Aseptis binotata* (Walker, 1865)

**syn. *Aseptis paviae* (Strecker, 1874)**

**syn. *Aseptis genitrix* (Grote, 1878)**

**syn. *Aseptis genetrix*, misspelling of authors, not Grote, 1878**

**syn. *Aseptis dilara* (Strecker, 1899)**

**syn. *Aseptis bultata* (Smith, 1906)**

**syn. *Aseptis cara* (Barnes & McDunnough, 1912)**

**932648.1 *Paraseptis****adnixa* (Grote, 1880)

**syn. *Paraseptis pausis* (Smith, 1899)**

932749.1 ***Viridiseptis****marina* (Grote, 1874)

932756 *Stretchia****plusiiformis*** H. Edwards, 1874

syn. *Stretchia plusiaeformis* H. Edwards, 1874, invalid emmendation

932810 *Nephelodes minians* Guenée, 1852

**syn. *Nephelodes mendica* Barnes & Lindsey, 1921**

**syn. *Nephelodes adusta* Buckett, 1973**

**932823.1 *Anarta melanopa* (Thunberg, 1791)**

932928 *Dargida diffusa* (Walker, 1856)

**syn. *Dargida terrapictalis* (Buckett, 1969)**

932934 *Mythimna****yuconensis*** (Hampson, 1911)

932938.1 ***Leucania amygdalina* (Harvey, 1878)**

933038 *Lacinipolia leucogramma* (Grote, 1873)

**syn. *Lacinipolia canities* (Hampson, 1905)**

**syn. *Lacinipolia francisca* (Smith, 1910)**

**933042.1 *Lacinipolia acutipennis* (Grote, 1880)**

**syn. *Lacinipolia subalba* Mustelin, 2000**

**933042.2 *Lacinipolia sareta* (Smith, 1906)**

**933042.3 *Lacinipolia dimocki* Schmidt, 2015**

933049 **Delete***Lacinipolia perta* (Druce, 1889)

**933115.1 *Protorthodes texicana* Lafontaine, 2014**

**933115.2***Protorthodes perforata* (Grote, 1883)

**syn. *Protorthodes constans* (Dyar, 1918)**

**933115.3***Protorthodes rufula* (Grote, 1874)

**933115.4 *Protorthodes ustulata* Lafontaine, Walsh & Ferris, 2014**

**933115.5***Protorthodes alfkenii* (Grote, 1895)

**933115.6***Protorthodes antennata* (Barnes & McDunnough, 1912)

933116 ***Nudorthodes****texana* (Smith, 1900)

933117 ***Nudorthodes****variabilis* (Barnes & McDunnough, 1912)

933117.1 ***Nudorthodes molino*** Walsh, Lafontaine & Ferris, 2014

**933331.1 *Euxoa bivittata* Lafontaine, 1987**

## Notes

p. 3 & p. 42 **Subfamily Dyopsinae** – Zahiri et al. (2013) showed through DNA sequence results that a number of subfamilies and genera formerly associated with the Erebidae were basal lineages of the Noctuidae, including the subfamily Dyopsinae. Recent molecular results by Zahiri and associates show a close relationship between *Dyops* Guenée and *Litoprosopus* Grote. [Contributed by Reza Zahiri].

**930098 *Schizura ipomaeae*** – The original spelling of the species name is *ipomaeae*, the author stating that the species name is from the plant genus *Ipomaea*, an incorrect spelling of the plant genus *Ipomoea* L. However, according to the ICZN rules the original spelling should be maintained, unless there is internal evidence for a typographical error.

**930139 *Scotura annulata*** – This species was added to the North American list in Franclemont 1983 as *Zunacetha annulata* and listed that way by Lafontaine and Schmidt (2010). It was transferred to the genus *Scotura* following Miller (2009) in Lafontaine and Schmidt (2011). We did not notice at the time that Miller, in the 2009 publication, had reviewed the report of *Scotura annulata* in Texas and concluded there was no evidence to support the record. Following this, we remove the species from the check list.

**930142 *Gynaephora rossii*** – This species was originally described in the genus *Laria* Schrank, 1802, a junior homonym of *Laria* Scolipi, 1763 in the Coleoptera, so the author’s name and year of publication should be enclosed by parentheses. [Contributed by Lars Crabo].

**930196 *Propyria schausi*** – The origin of the supposed occurrence of this species in the United States traces a convoluted path through the historic literature. The syntype specimens of *schausi* came from the Henry Edwards collection, originating from Jalapa, Mexico. Two females, which are in the AMNH, were initially identified by Edwards as *Ptychoglene aequalis* Walker. Neumoegen and Dyar (1893) in their revision of North American Arctiinae reidentified the specimens as *Lycomorpha fulgens* (H. Edwards). Dyar (1898) later realized that these specimens were neither *Lycomorpha fulgens* nor *Ptychoglene aequalis*, and so described them as a new species, *Lycomorpha schausi*. Neumoegen and Dyar (1893) stated that *Lycomorpha fulgens* was distributed in Mexico and Arizona, but their concept of *Lycomorpha fulgens* must have included both true *Lycomorpha fulgens* of Arizona (very similar in facies to *Propyria schausi*) and the Mexican *Propyria schausi*. Dyar (1903) did not include *Propyria schausi* in his North American list. Hampson subsequently documented *Propyria schausi* from both Mexico and Arizona, but must have based this solely on Neumoegen and Dyar’s (1893) literature accounts as he states the species “...is unknown to me.” (Hampson 1898). The Arizona record of *Propyria schausi* can therefore be traced to Hampson’s oversight of Neumoegen and Dyar’s confusion of *Lycomorpha fulgens* and *Propyria schausi*. *Propyria schausi* disappeared from the North American checklists until Franclemont (1983) listed it again, possibly based on Hampson’s account. No U.S. specimens of *Propyria schausi* were located by us in the major museums, and Ferguson et al. (2000) did not document it. We therefore conclude that Franclemont (1983) either repeated Hampson’s error, or the record (re-)originated from a misidentification of another *Lycomorpha*. An additional complicating factor is its similarity to the zygaenid *Neoalbertia constans* (Edwards), which is identical in size, wing shape and coloration – but differs structurally, for example in the antennae, wing venation, and presence of chaetosoma on the head. For the above reasons, *Propyria schausi* is removed from the North American list.

**930217 *Manulea bicolor*** – This species was transferred from *Eilema* Hübner to *Manulea* Wallengren (subgenus *Setema* de Freina and Witt) by Dubatolov (2015).

**930227.1 *Crambidia xanthocorpa*** – This species, recently described from eastern United States, was previously included in the concept of *Crambidia cephalica* (Grote & Robertson, 1870) (Lewis 2014).

**930315 *Spilosoma pteridis*** – The name *Spilosoma danbyi* was informally raised from synonymy under *Spilosoma pteridis* to the status of a valid species by Ferguson et al. (2000). However, as already noted by Barnes and McDunnough (1917a), both *pteridis* and *danbyi* were described from Vancouver Island, BC and refer to the same taxon. The taxonomy of this group is complicated by extreme geographic (and local) variation in phenotypes and pronounced sexual dimorphism, but there is no morphological or molecular evidence that two different species (*pteridis* and “*danbyi*”) exist as depicted in Ferguson et al. (2000). *Spilosoma danbyi* is therefore revised back to the synonymy of *Spilosoma pteridis* (**stat. rev.**). Further work is needed to examine the relationship between *Spilosoma vagans* and *Spilosoma pteridis*, as it is possible that the latter is only a small, dark form of *Spilosoma vagans* that occurs in cooler, wetter climates.

**930350 *Hypocrita celina*** – Synonymy proposed by Becker (2013) and Vincent and Laguerre (2013).

**930358 *Dysschema howardi*** – Becker (2013) synonymized *howardi* (type locality: New Mexico) under *Dysschema thetis* (Klug, 1836), described from “Mexico,” but as pointed out by Laguerre et al. (2014), the large DNA barcode divergences in this group indicate that these taxonomic changes are not supported, and that there are multiple species under the name *thetis* (*sensu* Becker). Arizona specimens of *howardi* differ from two other taxa under the name *thetis* (one from Michoacan and one from Sinaloa and Jalisco) by at least 2.5% (DNA barcode sequence), and we therefore re-instate *howardi***stat. rev.** as a *bona species* until the group can be revised thoroughly.

**930392 *Neritos prophaea*** – This species was listed as *Neritos prophaea* by recent North American authors, unaware that Toulgoët (1992) had transferred *prophaea* to *Haemanota* Hampson. It was first listed for North America by Franclemont (1983) (as *Trichromia prophaea*), who indicated that it was possibly a stray. There are no known vouchers or other details. Ferguson et al. (2000) did not map this species, nor are there any specimens in the Franclemont collection at CUIC. Given the lack of supporting data and the fact that *Neritos prophaea* belongs to a complex of closely related Neotropical species, we delete *Haemanota prophaea* from the North American list until voucher specimens are secured.

**930402 *Cycnia collaris*** – *Cycnia tenerosa*, **syn. n.**, and *Cycnia inopinatus*, **syn. n.**, have previously been recognized as separate species based largely on phenotypic differences and geographic distribution. In particular, the color of the wings and thorax can vary from white to gray, and the patagia from yellow to gray. Comparison of genitalic structure and DNA barcode variation shows that gray and white phenotypes represent the same species, with gray versus white phenotypes prevalent in spring and summer, respectively. The holotype of *inopinatus* represents the gray phenotype (described from Indian River, Florida) and is here synonymized with *Cycnia collaris* (described from Mississippi). The name *Cycnia tenerosa* (described from Misantla, [Veracruz], Mexico) was applied to Texas and Florida specimens by Ferguson and Opler (2006), but as indicated above, western, southern and northern populations represent the same variable species. The *tenerosa* holotype (USNM; illustrated in Watson 1971) is structurally and phenotypically indistinguishable from the white form of *tenerosa*, and we therefore synonymize the two.

**930440 *Cisseps fulvicollis*** – *Cisseps packardii* (Grote, 1865) and *Cisseps wrightii* (Stretch, 1885) were thought to be disjunct West Coast species related to the widespread *Cisseps fulvicollis*, differing from that species by a paler yellow scape (*packardii*) and slightly smaller size (*wrightii*). It is now evident that scape color and size varies in many western populations, and the geographic distribution is more continuous than historical workers realized. Maximum DNA barcode variation among all three taxa is less than 0.5%. We therefore treat *packardii*, **syn. n.** and *wrightii*, **syn. n.** as synonyms of *Cisseps fulvicollis*.

**930445 *Eucereon erythrolepsis*** – The spelling *erythrolepis* is an unjustified emendation of *erythrolepsis* introduced by Hampson (1914) and repeated in Ferguson and Opler (2006), Schmidt and Opler (2008) and Lafontaine and Schmidt (2010).

**930457 *Cosmosoma teuthras*** – Described from Venezuela, this taxon was first listed for the North American fauna by Barnes and McDunnough (1917b); however, there are no details of any records or vouchers. Because *Cosmosoma teuthras* is often confused with other *Cosmosoma*, we remove it from the North American list.

**930462.1 *Syntomeida syntomoides*** – Recorded in 2013 from Bahia Honda, Florida by Jim Troubridge. Vouchers in JTTC and CNC.

**930581 *Hypena tenebrosa*** – The barcodes were the first clue that *Hypena tenebrosa* (Walker, 1865), described from Venezuela, is conspecific with *Hypena annulalis* (Grote, 1876), **syn. n.**, described from Texas. They share similar forms throughout the species range.

**930604 *Rusicada privata*** – In our check list (Lafontaine and Schmidt 2010) we retained a broad concept of the genus *Anomis* Hübner on the basis that Holloway’s (2005) generic rearrangement for the Old World *Anomis* s.l. fauna excluded the New World taxa, accepting that a separate revision of New World generic limits in *Anomis* s.l. was needed first. However, the species *Anomis commoda* (Walker, 1865) is an introduced species that belongs to the Old World genus *Rusicada* Walker, so it seems reasonable to accept this generic transfer for consistency between Old World and New World names for shared species.

**930605 *Anomis impasta*** – This species has remained on the North American list since it was added by Kimball (1965). Even then, the identity of a Floridian taxon was suspected to not be correctly associated with *Anomis impasta*, which was described from French Guiana. Comparison of specimens from Florida with the lectotype of the species in MNHN, Paris, confirms that the taxon in Florida is not *Anomis impasta*, so it is hereby referenced as *Anomis impasta* of authors, not Guenée, 1852.

**930616 *Gonodonta fulvangula*** – The species name was misspelled as *fulvangala* in Lafontaine and Schmidt 2010.

**930631 *Psammathodoxa cochlidioides*** – Examination of the type specimen of *Sudariophora calligrapha* Hampson, 1926 from Brownsville, Texas, shows it to be the same species as *Psammathodoxa cochlidioides*. The species was transferred to the genus *Phyprosopus* Grote when *Sudariophora* Zeller was placed into its synonymy, the two generic names sharing the same type species. Therefore, *Sudariophora calligrapha* Hampson, 1926 is now transferred to *Psammathodoxa* Dyar as *Psammathodoxa calligrapha* (Hampson, 1926), **comb. n.**, **syn. n.**

**930703 *Hemeroplanis reversalis*** – Examination of the type specimens of *Hemeroplanis reversalis* (type locality: Brownsville, Texas) and *Hemeroplanis cuernavacalis* (Dyar, 1912), **syn. n.** (type locality: Cuernavaca, Mexico) shows them to be the same species.

**930710 *Hemeroplanis immaculalis*** – The types of *Hemeroplanis immaculalis* and *Hemeroplanis obliqualis*, **syn. n.**, represent the same species. Previously, the name *Hemeroplanis immaculalis* was applied to pale specimens of *Hemeroplanis parallela* (Smith, 1907) from Texas. Poole (1989) incorrectly credits Morrison rather than Edwards as the author of the name and gives the type repository as MSU, whereas the holotype is in AMNH.

**930727 *Hyperstrotia nana*** – Two nominal species are confused in collections, *Hyperstrotia nana*, described from Florida by Hübner in 1818, and *Hyperstrotia aetheria*, described by Grote from Florida in 1879. The type specimen of *Hyperstrotia aetheria* is in the BMNH, however there is no type specimen for *Hyperstrotia nana*, so the identity of the name is based on the painting in Hübner 1818, which is a new senior synonym of *Hyperstrotia villificans* (Barnes & McDunnough, 1918), **syn. n.** Another species of *Hyperstrotia* in Florida related to *Hyperstrotia aetheria* remains undescribed.

**930769.1 *Catocala myristica*** – New species from Kons and Borth (2015a).

**930846.1 *Catocala aestivalia*** – New species from Kons and Borth (2015b).

**930907 *Drasteria maculosa*** – This name represents populations of *Drasteria* in the Sierra Nevada that may be disjunct populations of *Drasteria hudsonica* (Grote & Robinson) or a closely related species. However, the name *Drasteria nubicola* (Behr, 1870), **syn. n.**, represents a pale almost unmarked form of the Sierran taxon. The name *Drasteria nubicola* has page priority over *Drasteria maculosa* (by one page), but as first revisers we use the name *maculosa* for the taxon because it represents the more typical form for the *Drasteria hudsonica* group.

**930925.1 *Doryodes desoto*** – New species from Lafontaine and Sullivan (2015).

**930925.2 *Doryodes okaloosa*** – New species from Lafontaine and Sullivan (2015).

**930927 *Doryodes spadaria*** – Revised synonymy of *Doryodes grandipennis* with *Doryodes spadaria* from Lafontaine and Sullivan (2015).

**930927.1 *Doryodes fusselli*** – New species from Lafontaine and Sullivan (2015).

**930927.2 *Doryodes latistriga*** – New species from Lafontaine and Sullivan (2015).

**930927.3 *Doryodes broui*** – New species from Lafontaine and Sullivan (2015).

**930927.4 *Doryodes reineckei*** – New species from Lafontaine and Sullivan (2015).

**930970.1 *Lesmone aenaria*** – This species was described from Mexico and Guatemala as *Bendis aenaria* and until now has been treated as a synonym of *Lesmone detrahens*. However, both the barcodes and numerous differences in the male genitalia show that *Lesmone aenaria*, **stat. rev.**, should be treated as a valid species distinct from *Lesmone detrahens*, the latter being widespread through eastern United States. *Lesmone aenaria* is known to occur from Costa Rica northward to southern Texas. The name *Metalectra lamprea* Druce, 1890, **syn. rev.**, described from Costa Rica, is a synonym of *Lesmone aenaria*, not a synonym of *Lesmone detrahens*.

**930982 *Heteranassa mima*** – New synonymy with *Heteranassa fraterna* and *Heteranassa minor* from Homziak et al. 2015.

**931000 *Toxonprucha excavata*** – There are at least four species in southern United States that have been collectively associated with the name *Toxonprucha diffundens* (Walker, 1858). However the holotype of *Celaena diffundens* Walker, and its synonym Pyralis? noctualis Walker, [1866], both described from Venezuela, differ significantly in ways that suggest this species may not be correctly placed in the genus *Toxonprucha* Möschler (1890) [Type species: *Toxonprucha amoena* Möschler, 1890; type locality: Puerto Rico], and certainly not conspecific with any of the synonyms or specimens from other areas that have been associated with the name. In “Toxonprucha” diffundens the hindwing is mainly pale and unpatterned, with some fuscous along the margin in both sexes, and the forewing pattern is reduced with the postmedial line forming an almost complete loop around the reniform spot, and the antemedial line forming a double-sided loop projecting from the costa, suggesting that *Celaena diffundens* does not belong in the genus *Toxonprucha*. We apply the name *Toxonprucha excavata* (Walker, 1865), **stat. rev.**, with its synonyms *Toxonprucha minuscula* (Walker, 1865), **syn. n.**, and *Toxonprucha amoena* Möschler, 1890, **syn. n.**, to populations that occur in southern Texas as suggested by similarity in barcodes from the Caribbean, Mexican, and Texan populations.

**931018 *Zale viridans* (Guenée, 1852)** – Specimens from Florida identified as *Zale viridans* (Guenée, 1852) are an undescribed species in the *Zale strigimacula* species complex and are not closely related to *Zale viridans*.

**931019 *Zale strigimacula* (Guenée, 1852)** – Specimens from Florida identified as *Zale strigimacula* (Guenée, 1852) represent the same species in the *Zale strigimacula* species complex as those misidentified as *Zale viridans*.

**931062 *Amolita sentalis*** – *Amolita sentalis* was doubtfully added to the North American list by Kimball (1965) on the basis of material in the AMNH identified as this species by Grossbeck (1917). However, Grossbeck, in his list of the Lepidoptera of Florida, did not list *Amolita obliqua* Smith, 1903, which was described from Florida. Therefore, it seems more likely that Grossbeck was following the synonymy of the two names by Hampson (1901) rather than recognizing the occurrence of *Amolita sentalis* as a species distinct from *Amolita obliqua*. Kimball (1965) states that *Amolita sentalis* is much smaller than *Amolita obliqua*; however, it appears that there is too much variation in size for this to be used as an identification character. *Amolita obliqua* and *Amolita sentalis* [TL: Trinidad] cannot be identified by external characters, only by genital characters, which are diagnostically different.

**931096 –931100 *Litoprosopus*** – The genus *Litoprosopus* Grote has for many years been classified among the “quadrifid noctuids”, now the family Erebidae. Lafontaine and Schmidt (2010) placed them near the end of the Erebidae in the subfamily Eulepidotinae as “*insertae sedis* Group 2”. Recent nuclear gene research by Reza Zahiri has determined that, like several other lineages formerly classified in the Erebidae (Zahiri et al. 2013), *Litoprosopus* should correctly be included in the basal Noctuidae in the subfamily Dyopsinae. Accordingly, the subfamily Dyopsinae is inserted into the check list on page 42 after the heading “**Family Noctuidae**” and the check list numbers for the five species are changed to 931160.1 to 931160.5. [Contributed by Reza Zahiri].

**931111 *Paectes abrostoloides*** – *Paectes delineata* (Guenée, 1852), **syn. n.**, has been treated as a valid species, mainly because there is no type material to deduce the synonymy. Like several other species described by Guenée, the description likely was based on a painting by John Abbot in the late 1700’s in the Savannah, Georgia area. Given that the description matches one of the known forms of *Paectes abrostoloides*, and no other similar species occur in the area, we treat *Paectes delineata* as a new synonym of *Paectes abrostoloides*.

**931156 *Collomena inflexa*** – The species that occurs in Florida is *Collomena inflexa* (Morrison, 1875), **stat. rev.** (Type-locality: Jacksonville, Florida). It was treated as a synomym of *Collomena filifera* (Walker, 1857) (Type-locality: Santo Domingo, Dominican Republic). The two species differ in the configuration of the eversible antler-like coremata on the valves, and by 2.5% in their barcode sequences.

**931253 *Amyna stricta*** – Barcoding was the original clue that New World populations of *Amyna stricta* (Walker, 1858), **stat. rev.**, are not conspecific with Old World populations, known as *Amyna axis* Guenée, 1852 [= *Amyna octo* Guenée, 1852]. Consistent differences in the pouches and spine clusters in the vesica support the separation of Old and New World populations as separate species. *Amyna flaviguttata* (Walker, 1858), **syn. rev.** [Type locality: Venezuela], *Amyna orbica* (Morrison, 1874), **syn. rev.** [Type locality: Texas], and *Amyna tecta* (Grote, 1876), **syn. rev.** [Type locality: Texas], are synonyms of *Amyna stricta* [Type locality: Honduras].

**931253.2 *Concana mundissima*** – Nuclear gene research on the basal lineages of the Noctuidae (Zahiri et al. 2013) resulted in the genus *Concana* Walker being moved from the Nolidae, Collomeninae to the Noctuidae, Bagisarinae.

**931254.1 *Cydosia majuscula*** – Barcode differences confirm that *Cydosia majuscula* (H. Edwards, 1881), **stat. rev.**, the all black “form” of *Cydosia aurivitta* Grote & Robinson, 1868, is a valid species confined to southeastern United States east of Texas. It was described from Georgia [type in AMNH]. *Cydosia aurivitta*, the “maculate form” is a separate species that occurs in Texas and Mexico. It occurs in two basic forms, one with black forewings and broad reddish-orange transverse bands and a similarly-colored square in between the two lines, and a second form covered with large white spots in addition to the orange bands and spot. *Cydosia aurivitta* and its synonym *Cydosia imitella* Stretch were described from Texas; the types are in ANSP and USNM respectively. [Contributed by Hugo Kons, Jr., Gainesville, Florida].

**931257.1 *Tripudia inquaesita*** – Barcode differences and details in maculation show that the species of *Tripudia* Grote in the *Tripudia damozela* complex (*Tripudia inquaesita* (Barnes & Benjamin, 1924), **stat. rev.**) is a distinct species and occurs in southern Arizona and probably in the Sierra Madre Occidental in Mexico, and is different from *Tripudia damozela*, which occurs from the State of Puebla in southeastern Mexico northward in the Sierra Madre Oriental to western Texas.

**931412 *Raphia frater*** – Revisions in synonymy and subspecies ranking from Schmidt and Anweiler (2014).

**931442.1 *Acronicta fallax*** – Generic combination, placement, and synonymy (syn. *Acronicta geminata* (Smith, 1903)) from Schmidt et al. (2014).

**931477.1 *Acronicta menyanthidis*** – This species was first recorded for North America in Ferris et al. (2012). So far, it has only been found in Alaska.

**931496 *Chloronycta tybo*** – The genus *Chloronycta* Schmidt & Anweiler, and the generic combination, were proposed in Schmidt et al. (2014).

**931550 *Psaphida rolandi*** – This species was described the in the genus *Eutolype* Grote, so parentheses should enclose the author and date to reflect the generic transfer.

**931564 *Feralia comstocki*** – This species was described in the genus *Feralia* Grote, so the parentheses around the author and date should be removed.

**931606 *Emarginea percara*** – The species formerly known as *Emarginea pallida* (Smith, 1902), **syn. n.**, is redefined as a very pale desert form of *Emarginea percara* known from the deserts of southeastern California, southernmost Nevada, and western Arizona. The specimens are almost devoid of the black lines that define the forewing maculation in the typical form, but they differ neither in genitalia nor barcodes from specimens of *Emarginea percara* from other areas.

**931642 *Cropia connecta*** – Rearing of *Cropia connecta* in Costa Rica (Janzen and Hallwachs 2015) and barcode results show that *Cropia templada* (Schaus, 1906), **syn. n.**, is a form of *Cropia connecta*.

**931643.1 *Cropia indica*** – Specimens of this Caribbean species collected on Key Largo, Florida are in the collections of CUIC, TLSC, and USNM. They were collected the same year so it may have been a temporary incursion.

**931767 *Catabenoides vitrina*** – Kimball (1965) recognized two species in southern Florida, *Catabenoides vitrina* [Type locality: Dominican Republic] and *Catabenoides divisa* [Type locality: Cuba] based on unpublished data credited to E. Todd. There are two species in southern Florida, one with a black streak in the female from the base of the forewing to just below the apex, and one with an entirely gray female. However, it is clear from the original descriptions that both names refer to the same species, so *Catabenoides divisa* (Herrich-Schäffer, 1868), **syn n.**, is the same species as *Catabenoides vitrina* and the second species in Florida is undescribed.

**931773 *Pseudacontia crustaria*** – This is an uncommon species of the Great Plains and few specimens have been available for study. It occurs in three forms, a more northerly form with a mainly white forewing with dark shading in the basal, outer half of the subterminal, and the terminal areas (*Pseudacontia crustaria*), a form with dark shading in the basal, medial and terminal areas (*Pseudacontia louisa***syn. n.**), and a mainly dark form with a pale band between the basal and medial areas, and in the outer part of the medial area (*Pseudacontia cansa***syn. n**). There are no differences in the genitalia nor barcodes.

**931784 *Stylopoda groteana*** – Smith (1908) described ***Stylopoda anxia***, **syn. n.** from Ft Wingate, New Mexico, apparently unaware that Dyar (1903) described it from Williams, Arizona, as *Pseudacontia groteana* five years previously. There are no characters to support the recognition of two species in the range of *Stylopoda groteana*, which occurs from western New Mexico eastward through Arizona and southern Utah to the New York Mountains in eastern California. [Contributed by David Wikle].

**931906.1 *Sympistis forbesi*** – A new species related to *Sympistis chionanthi* (J.E. Smith, 1797) described in Zacharczenko et al. 2014.

**932022.1 *Ogdoconta margareta*** – New species from Crabo (2015).

**932036 *Heliothodes diminutivus*** – The species name was misspelled as *diminutiva* and synonym ***fasciata*** misspelled as *fasciatus* in Lafontaine and Schmidt 2010.

**932044 *Psectrotarsia hebardi*** – The species name described in the genus *Erythroecia* Hampson, so the author and date should be placed in parentheses. [Contributed by Lars Crabo].

**932054 *Chloridea virescens*** – Generic combination from Pogue (2013).

**932055 *Chloridea subflexa*** – Generic combination from Pogue (2013).

**932223 *Spodoptera eridania*** – The correct authorship and date for this species is Stoll, 1782 (Poole 1989). The confusion between the authorship of Cramer and Stoll was discussed by us previously (Lafontaine and Schmidt 2011: 150).

**932236.1 *Elaphria trolia*** – *Elaphria trolia* (Dyar, 1914), **stat. n.**, was described and previously treated as a Central American subspecies of *Elaphria exesa* (Guenée, 1852), but differs from it in barcodes (3.5%) and male genital characters. So, it is revised to the status of valid species. [Contributed by Jim Troubridge].

**932278 *Acroria pulchra*** – Barcode results show that *Acroria terrens* (Walker, 1857) [TL: Venezuela], and its synonyms *Acroria infensa* (Walker, 1857) [TL: Venezuela], and *Acroria villipes* Walker, 1858 [TL: Brazil], is a different species from *Acroria pulchra* (Möschler, 1886), **stat. rev.**, [TL: Jamaica], and *Acroria pulcha*’s synonym *Acroria niphanda* (Druce, 1889) [TL: northern Panama], **syn. rev.***Acroria terrens* was barcoded from Venezuela, Brazil, Saint Lucia, and Puerto Rico. *Acroria pulchra* was barcoded from Costa Rica, Guatemala, Mexico, USA, Texas, Dominican Republic, and Jamaica.

**932284 *Nedra stewarti*** – The species *Nedra dora* was described as being similar to *Nedra stewarti*, but with the forewing a darker purplish fuscous with lighter lavender-gray area than *Nedra stewarti*. There is a great deal of variation in color from place to place and the genital characteristics given by Clarke (1940) do not hold up when more material is examined. Also, the barcodes are not differentiated from those of *Nedra stewarti*, So we place *Nedra dora* Clarke, 1940, **syn. n.** in synonymy.

**932361.1 *Cherokeea attakullakulla*** – The genus *Cherokeea* Quinter & Sullivan, 2014, and the species *Cherokeea attakullakulla* Sullivan & Quinter, 2014, were described in Quinter and Sullivan 2014.

**932480 *Papaipema insulidens*** – *Papaipema insulidens* was described from Vancouver Island in southwestern British Columbia; *Papaipema pertincta* Dyar, 1920, **syn n.**, was described from Forest Grove in northwestern Oregon; *Papaipema birdi* (Dyar, 1908), **syn n.**, was described from Rye, New York. We treat *Papaipema insulidens* as a widespread species that ranges across the southern boreal zone of Canada to connect the eastern and western parts of its range. Throughout this vast area specimens can range from a pale grayish yellow to a darker reddish brown with forms covering this variation sometimes from the same localities. There are no structural differences throughout this range and the barcodes are the same with one haplotype shared almost through the whole range from North Carolina to the Pacific Coast. For these reasons we treat *Papaipema pertincta* and *Papaipema birdi* as synonyms of *Papaipema insulidens*.

**932483 *Papaipema harrisii*** – The taxon *Papaipema verona*, described from Winnipeg, Manitoba, Canada, refers to prairie populations of the species otherwise known from eastern North America as *Papaipema harrisii*. The specimens are indistinguishable from those of *Papaipema harrisii* from farther east in appearance, genitalia, and barcodes, so we treat *Papaipema verona* (Smith, 1899), **syn. n.**, as a synonym of *Papaipema harrisii*.

**932552 *Lithophane viridipallens*** – The species *Lithophane pruena***syn. n.**, was described by Dyar (1910) from a single female from eastern Texas. The type specimen is lost, but *Lithophane pruena* is described as being similar to *Lithophane viridipallens*, but the basal dash in *Lithophane pruena* is more prominent, there is more black shading between the reniform and orbicular spots, and the dark spots along the subterminal line are larger than in typical specimens of *Lithophane viridipallens*. A specimen in the TLSC matches the original description of *Lithophane pruena* perfectly and its barcodes are identical to those of *Lithophane viridipallens* from Georgia, North Carolina and New Jersey. Because there is considerable variation in the diagnostic features of *Lithophane viridipallens*, we treat *Lithophane pruena* as a well-marked form of it. Although the type specimen is lost, the female genitalia slide of the type of *Lithophane pruena* is in USNM and is indistinguishable from those of other *Lithophane viridipallens*.

**932634 *Aseptis binotata*** – Revision of the genus *Aseptis* McDunnough, 1937, by Mustelin and Crabo (2015) resulted in *Aseptis paviae* (Strecker, 1874), *Aseptis genitrix* (Grote, 1878), *Aseptis dilara* (Strecker, 1899), *Aseptis cara* (Barnes & McDunnough, 1912), and *Aseptis bultata* (Smith, 1906), being placed into synonymy with *Aseptis binotata* as geographical forms. Mustelin and Crabo (2015) point out that the name *Aseptis genitrix* was misspelled as *Aseptis genetrix* by numerous authors including Franclemont and Todd (1983), Poole (1989), and Lafontaine and Schmidt (2010).

**932648.1 *Paraseptis adnixa*** – In a revision of the genus *Aseptis*, Mustelin and Crabo (2015) proposed a new genus, *Paraseptis* Mustelin & Crabo, 2015, for *Hadena adnixa*, a species formerly included in *Aseptis*. In their revision, the species *Aseptis pausis* (Smith, 1899) is synonymized with *Paraseptis adnixa* as *Paraseptis pausis*.

**932749.1 *Viridiseptis marina*** – The genus *Viridiseptis* Mustelin & Crabo, 2015, was proposed in Mustelin and Crabo (2015) for the species previously listed as 932649 *Aseptis marina* (Grote, 1874) in Lafontaine and Schmidt (2010). The species is highly divergent from other genera in the Xylenini, therefore Mustelin and Crabo (2015) recommended it be moved to Xylenini*incertae sedis*; this change in position is reflected in its revised check list number.

**932756 *Stretchia plusiiformis*** – *plusiaeformis* is an unjustified emendation of the original spelling *plusiiformis*. Incorrect latinization of a name is not to be considered an incorrect original spelling (ICZN 1999, Article 32.5.1).

**932810 *Nephelodes minians*** – On the basis of variability in genital characters, geographical variation, and shared barcode sequences, we treat *Nephelodes mendica* Barnes & Lindsey, 1921, **syn. n.**, and *Nephelodes adusta* Buckett, 1973, **syn. n.**, as part of the geographical variability of *Nephelodes minians*.

**932823.1 *Anarta melanopa*** – *Anarta nigrolunata* Packard, 1867, was segregated from its Old World sister species by Schmidt and Anweiler 2010. The two species can be distinguished by genital characters and barcode sequences. Recently, specimens of *Anarta melanopa* were barcoded from Alaska and northern Yukon and represent new records of this species in North America. Curiously, the barcodes are identical with those from specimens from montane areas of southern Europe, but differ from those from Fennoscandia, suggesting a southern Siberian-Altai-Balkans-Alps connection to the Alaskan population, not a northern Russia-Fennoscandian connection as is seen in more arctic-occurring species like *Sympistis zetterstedtii* (Staudinger, 1857).

**932928 *Dargida diffusa*** – Lack of consistent genital differences, identical barcodes with those of *Dargida diffusa* from various parts of Canada and the United States, and intermediate forms in Idaho and western Montana, result in us treating *Dargida terrapictalis* (Buckett, 1969), **syn. n.**, as conspecific with *Dargida diffusa*. *Dargida terrapictalis* is a dark form of the species that occurs from southern British Columbia to central California and could be treated as a subspecies if desired.

**932934 *Mythimna yuconensis*** – We return this species to its original spelling. The name was emended by McDunnough (1938) to *yukonensis*, not accepting Hampson’s use of a c in the name instead of a k. The ICZN rules allow the original spelling of a name to be “corrected” if the person or place the name is derived from is included in the original text, thus demonstrating the error. However, in the case of *yuconensis*, it was Hampson’s habit to use traditional Latin letters for scientific names, including changing the species name in *Xestia wockei* to *voccei*. The spelling of *yuconensis* cannot be taken as an error, but was the spelling intended by Hampson.

**932938.1 *Leucania amygdalina*** – Barcode results, and consistent differences in the genitalia between southern populations of *Leucania linita* [type-locality: St. John’s Bluff, Florida] and those from farther north result in us treating the northern populations as *Leucania amygdalina* (Harvey, 1878), **stat. rev.**, [type locality: Orono, Maine] as a valid species. *Leucania linita* has a longer, more coiled vesica and a larger cucullus (40% as long as saccular region) in the male genitalia, whereas the vesica is shorter and straighter and the cucullus is 30% as long as the saccular region in *Leucania amygdalina*. *Leucania linita* is known to occur from Maryland to Florida and Texas; *Leucania amygdalina* occurs from southern Canada and New England westward to Illinois and Indiana and southward probably to New Jersey.

**933038 *Lacinipolia leucogramma*** – Two names formerly treated as valid species, *Lacinipolia canities* (Hampson, 1905), **syn. n.** and *Lacinipolia francisca* (Smith, 1910), **syn. n**., are synonyms of *Lacinipolia leucogramma*. This result was reported in the unpublished 1975 thesis of Charles Selman, and the barcodes results also support this conclusion.

**933042.1 *Lacinipolia acutipennis*** – New synonymy from Schmidt (2015).

**933042.2 *Lacinipolia sareta*** – Revised status from Schmidt (2015).

**933042.3 *Lacinipolia dimocki*** – New species from Schmidt (2015).

**933049 *Lacinipolia perta*** – This Mexican species was added to the North American by Franclemont and Todd (1983), presumably from material collected by Franclemont in southern Arizona. Specimens of *Lacinipolia lepidula* (Smith, 1888) from the southwestern United States with a darker medial area are a good match for the type of *Lacinipolia perta*, described from Veracruz, Mexico. However, other species from southeastern Mexico, such as *Lacinipolia perfragilis* (Dyar, 1923), also added to the MONA list by Franclemont and Todd (1983), and *Abagrotis totonaca* (Schaus, 1894), have been shown to be similar but distinct species from their sister species in southwestern United States and the Sierra Madre Occidental in Mexico (Lafontaine and Schmidt 2010: 165; Lafontaine 1998: 217). For these reasons, we remove *Lacinipolia perta* from the list of North American Noctuidae north of Mexico. The type material of *Lacinipolia perta*, was not located in the BMNH in 2007 or 2010 (JDL), therefore we do not know if *Lacinipolia perta* is a valid species, or a synonym of *Lacinipolia lepidula*.

**933115.1 *Protorthodes texicana*** – Described by Lafontaine (2014) in Lafontaine et al. (2014).

**933115.2 *Protorthodes perforata*** – Syn. *Protorthodes constans*; synonymy from Lafontaine et al. (2014).

**933115.3 *Protorthodes rufula*** – Species renumbered to reflect change in phylogenetic position in Lafontaine et al. (2014).

**933115.4 *Protorthodes ustulata*** – New species from Lafontaine et al. (2014).

**933115.5 *Protorthodes alfkenii*** – Species renumbered to reflect change in phylogenetic position from Lafontaine et al. (2014).

**933115.6 *Protorthodes antennata*** – Species renumbered to reflect change in phylogenetic position from Lafontaine et al. (2014).

**933116 *Nudorthodes texana*** – Generic description and new combination from Lafontaine et al. (2014).

**933117 *Nudorthodes variabilis*** – New combination from Lafontaine et al. (2014).

**933117.1 *Nudorthodes molino*** – New species from Lafontaine et al. (2014).

**933331.1 *Euxoa bivittata*** – Described as a subspecies of *Euxoa vallus* (Smith, 1900); however, barcode results, and male genital characters lead us to treat *Euxoa bivittata* Lafontaine, 1987, **stat. n.**, as a valid species. Superficially, the moths differ from those of *Euxoa vallus* in having broad, fuzzy, almost straight, transverse lines, whereas in *Euxoa vallus* the antemedial line is zigzagged and the medial line is serrated and bends basally near the middle of the wing to pass between the reniform and orbicular spots. The vesica in *Euxoa bivittata* has a stronger sub-basal twist, so the apical part of the vesica is farther above the basal part than in *Euxoa vallus*, and the sub-basal diverticulum follows the direction of the aedeagus more closely. Curiously, the barcodes of *Euxoa vallus* are more similar to those of *Euxoa macleani* McDunnough than to those of *Euxoa bivittata*.
